# Brain Myelin Covariance Networks: Gradients, Cognition, and Higher-Order Landscape

**DOI:** 10.3390/bs15111466

**Published:** 2025-10-28

**Authors:** Huijun Wu, Arpana Church, Xueyan Jiang, Jennifer S. Labus, Chuyao Yan, Emeran A. Mayer, Hao Wang

**Affiliations:** 1 School of Media & Communication, Shanghai Jiao Tong University, Shanghai 200240, China; 2 G. Oppenheimer Center for Neurobiology of Stress & Resilience, University of California Los Angeles (UCLA), Los Angeles, CA 90095, USA; 3 Vatche and Tamar Manoukian Division of Digestive Diseases, Department of Medicine, David Geffen School of Medicine, University of California Los Angeles (UCLA), Los Angeles, CA 90095, USA; 4 Goodman-Luskin Microbiome Center, David Geffen School of Medicine, University of California Los Angeles (UCLA), Los Angeles, CA 90095, USA; 5 State Key Laboratory of Digital Medical Engineering, Key Laboratory of Biomedical Engineering of Hainan Province, School of Biomedical Engineering, Hainan University, Sanya 572025, China; 6 School of Psychology, Nanjing Normal University, Nanjing 210097, China

**Keywords:** cognitive functions, myelin covariance networks, gradient analysis, vertex-level, neurological disorders

## Abstract

Myelin is essential for efficient neural signaling and can be quantitatively evaluated using the T1-weighted/T2-weighted (T1w/T2w) ratio as a proxy for regional myelin content. Myelin covariance networks (MCNs) reflect correlated myelin patterns across brain regions, enabling the investigation of topological organization. However, a vertex-level map of myelin covariance gradients and their cognitive associations remains underexplored. The objective of this study was to construct and characterize vertex-level MCNs, identify their principal gradients, map their higher-order topological landscape, and determine their associations with cognitive functions and other multimodal cortical features. We conducted a cross-sectional, secondary analysis of publicly available data from the Human Connectome Project (HCP). The dataset included T1w/T2w MRI data from 1096 healthy adult participants (age 22–37). All original data collection and sharing procedures were approved by the Washington University institutional review board. Our procedures involved (1) constructing a vertex-wise MCN from T1w/T2w ratio data; (2) applying gradient analysis to identify principal organizational axes; (3) calculating network connectivity strength; (4) performing cognitive meta-analysis using Neurosynth; and (5) using graphlet analysis to assess higher-order topology. Our results show that the primary myelin gradient (Gradient 1) spans from sensory-motor to association cortices, strongly associates with connectivity strength (r = 0.66), and shows a functional dissociation between affective processing and sensorimotor domains. Furthermore, Gradient 2, as well as the positive and full connectivity strength, showed robust correlations with fractional anisotropy (FA), a DTI metric reflecting white matter microstructure. Our higher-order analysis also revealed that negative and positive myelin covariance connections exhibited distinct topologies. Negative connections were dominated by star-like graphlet structures, while positive connections were dominated by path-like and triangular structures. This systematic vertex-level investigation offers novel insights into the organizational principles of cortical myelin, linking gray matter myelin patterns to white matter integrity, and providing a valuable reference for neuropsychological research and the potential identification of biomarkers for neurological disorders.

## 1. Introduction

The human brain exhibits intricate organization as a complex system (Kulkarni & Bassett, 2025; Presigny & De Vico Fallani, 2022). Myelin, an important microstructural feature of the nervous system, supports efficient axonal signal transmission and strongly impacts brain structure connectivity (Fotiadis et al., 2023; Glasser & Van Essen, 2011; Schaffner et al., 2023; van der Weijden et al., 2021). Structural covariance, or, the interregional correlation of morphological measurements at the population level, is an important biomarker that allows for the investigation of brain connectivity architectures and neurodevelopmental trajectories (Alexander-Bloch et al., 2013; He et al., 2007; Sebenius et al., 2023). Structural covariance networks have become a widely adopted methodological framework for investigating both typical and atypical patterns of brain connectivity (Hunt et al., 2016; Ma & Zhang, 2017; Melie-Garcia et al., 2018; Vandewouw et al., 2021), given that structural covariance networks can be created from interregional statistical correlations in morphometric measures (i.e., gray matter volume, cortical thickness) or myelin proxies (i.e., T1w/T2w ratio) across brain regions (Alexander-Bloch et al., 2013). The T1w/T2w ratio has been validated as a reliable proxy for myelin, reflecting myelin content and microstructure (Du et al., 2019; Glasser & Van Essen, 2011; Wu et al., 2018). The co-variation of T1w/T2w ratio between brain regions across individuals may reflect their functional relationships (Hunt et al., 2016).

Although the majority of myelin group-level covariance networks utilize a region-of-interest (ROI) approach, to limit computational burden (Ma & Zhang, 2017; Melie-Garcia et al., 2018), vertex-wise analysis has several key advantages: (1) it preserves more information by not averaging features over a predefined ROI, thereby better maintaining individual variability (Revell et al., 2022); (2) it has greater spatial fidelity by not introducing arbitrary boundaries for ROI anatomy, and allowing for anatomical variability (Furtjes et al., 2023); (3) it has greater sensitivity to find small deviations in the morphology of myelin that could be aggregated when using a ROI-analysis approach (Hahn et al., 2022). This study utilized Human Connectome Project (HCP) data from 1096 individuals, a dataset widely used for studying brain connectivity and myelin (Glasser et al., 2016; Shen et al., 2017; Van Essen et al., 2013; Wu et al., 2024), employing the T1w/T2w ratio as a proxy for myelin to construct vertex-wise group-level covariance networks. The study aimed to produce vertex-wise group-level myelin covariance networks (MCN), and to characterize its topological structure, further incorporated gradient analysis (Nenning et al., 2024) and higher-order graphlet analysis (Przulj, 2007; Zitnik et al., 2024). Gradient analysis, a dimensionality reduction technique, revealed systematic axes of structural variation across the cortex, elucidating hierarchical relationships in myelin patterns across brain regions. Higher-order graphlet analysis extended beyond conventional node-edge paradigms by examining recurrent small subgraphs (graphlets) (Battiston et al., 2020), potentially reflecting specific neural circuit architectures and information processing pathways. The investigation advanced beyond basic myelin correlation measurements, demonstrating associations between principal gradients of vertex-level MCNs with network connectivity strength, cognitive domains (Coronel-Oliveros et al., 2025; Yarkoni et al., 2011), and the sensorimotor-association (S-A) axis (Pei et al., 2025; Sydnor et al., 2021). Furthermore, it moves past traditional low-order network analyses by employing graphlets to capture higher-order organizational patterns in myelin covariance dynamics (Battiston et al., 2020; Hočevar & Demčar, 2016; Przulj, 2007; Zitnik et al., 2024). By establishing detailed myelin covariance mapping, enhancing our understanding of how myelin shapes brain connectivity and higher-order network organization, potentially improving diagnostic and monitoring strategies for myelin-related neurological disorders (e.g., multiple sclerosis, Alzheimer’s disease) (Liu et al., 2017; J. Wang et al., 2013). These diseases are a major global health challenge. The prevalence of all-cause dementia (of which 60–70% is Alzheimer’s disease or mixed Alzheimer’s disease with other pathologies) was estimated at over 57 million people globally in 2021 (Frisoni et al., 2025), and multiple sclerosis impacts nearly 2.9 million (Ganjgahi et al., 2025). As myelin pathology is a core feature of these conditions (Bright et al., 2025; Cai et al., 2025; Matute & Verkhratsky, 2025; Vojdani et al., 2025), more sensitive biomarkers are urgently needed to monitor changes in cortical organization. The integration of vertex-wise covariance networks, gradient analysis, meta-analysis maps, and higher-order graphlets provides novel insights into the organizational principles of human brain myelin networks, offering vital perspectives on understanding the topological structure, gradient variations, cognitive associations, and higher-order features of myelin covariance networks, as well as future neuropsychological research.

The overall objective of this study was to construct and comprehensively characterize vertex-level myelin covariance networks (MCNs) using T1w/T2w ratio data from a large healthy adult sample. Our specific objectives were (1) to identify the principal organizational gradients of the MCN; (2) to investigate the relationships between these gradients, network connectivity strength, and cognitive functions (via Neurosynth meta-analysis) as well as other multimodal cortical features (e.g., anatomical hierarchy); and (3) to examine the higher-order topological landscape of the network using graphlet analysis. We hypothesized that (1) the principal myelin gradient would align with the established sensorimotor-association (S-A) axis, and (2) the network’s topological organization, particularly for positive and negative covariance networks, would exhibit distinct higher-order structural patterns reflecting the hierarchical organization of the human cortex.

## 2. Materials and Methods

### 2.1. Study Design

This study employed a cross-sectional, observational design, utilizing retrospective data from the publicly available Human Connectome Project (HCP) S1200 release. This research is not a diagnostic study; rather, its methodological objective is to construct and characterize the topological organization of vertex-level myelin covariance networks (MCNs) in a large, healthy adult sample. The study specifically aimed to identify principal gradients, map higher-order graphlet structures, and correlate these network features with cognitive domains and other multimodal cortical maps, as detailed in the following sections.

### 2.2. Participants

This study utilized MRI data from the Human Connectome Project (HCP, https://www.humanconnectome.org), a consortium led by Washington University, the University of Minnesota, and Oxford University (WU-Minn HCP) (Glasser et al., 2013; Van Essen et al., 2013). Data collected under the WU-Minn HCP is housed and distributed by the consortium. The HCP scanning protocol received approval from the Institutional Review Board at Washington University in St. Louis, and all participants provided informed consent. We downloaded the 1200 Subjects Group Average Data, released 1 August 2017 (https://www.humanconnectome.org/study/hcp-young-adult/article/s1200-group-average-data-release, accessed on 1 January 2024). Preprocessing and cleaning, detailed in the HCP S1200 release manual (https://www.humanconnectome.org/study/hcp-young-adult/document/1200-subjects-data-release, accessed on 1 January 2024), were performed using publicly available scripts (https://github.com/Washington-University/HCPpipelines, accessed on 1 January 2024).

For subsequent analysis, the *S1200.All.MyelinMap_BC_MSMAll.32k_fs_LR.dscalar.nii* dataset, containing individual myelin MSMAll-registered structural maps, was used. Exclusion criteria included a history of significant neurological, psychiatric, or medical disorders, substance abuse, or conditions contraindicating MRI scanning. Full details regarding participant recruitment and screening procedures are available in the HCP S1200 release manual and the consortium’s primary documentation (Van Essen et al., 2013). The study included 1096 participants (500 males, 596 females), with an average age of 28.78 years (age range: 22–37).

### 2.3. MRI Acquisition

All data were acquired using a 3 T Siemens Skyra MRI scanner (Siemens, Munich, Germany). T1-weighted structural images were obtained with the 3D magnetization-prepared rapid acquisition gradient echo (3D-MPRAGE) sequence, employing the following parameters: repetition time (TR) = 2400 ms, echo time (TE) = 2.14 ms, flip angle = 8°, field of view (FOV) = 224 × 224 mm^2^, voxel size = 0.7 × 0.7 × 0.7 mm^3^, acquisition time = 7 min 40 s, and BW = 210 Hz/Px. T2-weighted structural images were acquired using the 3D sampling perfection with application-optimized contrast using different flip-angle evolutions (3D-SPACE) sequence with the parameters: TR = 3200 ms, TE = 565 ms, FOV = 224 × 224 mm^2^, voxel size = 0.7 × 0.7 × 0.7 mm^3^, acquisition time = 8 min 24 s, and BW = 744 Hz/Px (Glasser et al., 2016; Glasser et al., 2013; Van Essen et al., 2013).

### 2.4. Image Preprocessing

Structural MRI data from the HCP were preprocessed using the HCP minimal preprocessing pipelines, encompassing PreFreeSurfer, FreeSurfer, and PostFreeSurfer (Glasser et al., 2013). Brain myelin content was estimated using the T1w/T2w ratio derived from magnetic resonance imaging (MRI) data. The HCP pipeline provides a standardized approach to preprocess HCP data and generate T1w/T2w myelin maps. This process utilizes the FreeSurfer software (version 5.3.0) suite to construct cortical surface representations (software available at https://surfer.nmr.mgh.harvard.edu). Subsequently, white matter, pial, and mid-thickness surfaces were mapped onto high-resolution Conte69 registered standard meshes, available in both ~164k and ~32k vertices per hemisphere. The HCP pipeline employs FSL’s FLIRT tool (version 5.0.6), using a mutual information cost function, to ensure optimal alignment between T1w and T2w maps. This alignment step is crucial because the subsequent division of the T1w image by the aligned T2w image specifically enhances cortical myelin contrast and reduces signal intensity biases. The theoretical basis of this approach is the relationship between the T1w/T2w ratio and myelin content. Myelin content (*m*) is approximately proportional to the T1w image intensity and inversely proportional to the T2w image intensity. Furthermore, both T1w and T2w images are affected by a common receive bias field (*b*). Consequently, the resulting T1w/T2w signal ratio shows an approximate proportionality to *m*^2^, effectively amplifying the myelin contrast-to-noise ratio. This ratio enhancement improves the signal-to-noise ratio for myelin mapping.

$$\frac{T1w}{T2w}\sim \frac{m*b}{(\frac{1}{m})*b}=m^{2}$$

### 2.5. Myelin Covariance Network Construction

In this study, we used the fsLR-32k myelin map for further analyses. To control confounding variables, we performed a linear regression on the myelin map to account for the effects of age, age-squared, gender, and age-gender interactions. Residuals from this regression were used for subsequent analysis. To quantify vertex-wise myelin covariance, we calculated the Pearson correlations of residual myelin content across all 1096 participants. This procedure yielded a vertex-wise myelin covariance matrix of size 59,412 × 59,412. The regression model is as follows

$$M_{v}=\beta_{0}+\beta_{1}\cdot \mathrm{age}+\beta_{2}\cdot {\mathrm{age}}^{2}+\beta_{3}\cdot \mathrm{gender}+\beta_{4}\cdot \left(\mathrm{age}\times \mathrm{gender}\right)+\epsilon_{v}$$

The residual of the model is expressed as $R_{v}=M_{v}-\hat{M_{v}}$, the Pearson correlation is calculated as

$$\rho_{ij}=\frac{\mathrm{cov}\left(R_{v_{i}},R_{v_{j}}\right)}{\sqrt{\mathrm{var}\left(R_{v_{i}}\right)\cdot \mathrm{var}\left(R_{v_{j}}\right)}}$$

### 2.6. Gradients of Vertex-Wise Myelin Covariance Network

Traditional methods for analyzing brain connectivity gradients at high resolution face enormous computational and memory demands. A recent study developed a novel approach called Fast Connectivity Gradient Approximation (FCGA) to reduce computational costs in connectome analysis while maintaining high spatial resolution (Nenning et al., 2024). By utilizing a set of landmarks to approximate connection architecture at full spatial resolution, FCGA avoids constructing complete vertex-to-vertex connectivity matrices, thereby significantly improving computational efficiency and reducing memory usage. Results demonstrate that FCGA preserves crucial individual features and enhances brain-behavior predictions, even in using 5% of vertices (≈3000), enabling broader application of connectivity gradients to capture spatial characteristics of brain connectomes. In implementation, FCGA was applied to compute gradients for the vertex-wise MCN using 3000 random landmarks, with the first three principal components extracted as main gradients.

### 2.7. Connectivity Strength of Vertex-Wise Myelin Covariance Network

To account for both positive and negative correlations (Saberi et al., 2021; Zhan et al., 2017) in the MCN, we separated the network into two distinct sub-networks based on the sign of the correlation values: one capturing negative correlations and the other capturing positive correlations. Importantly, no further thresholding was applied to either sub-network. For each vertex, we calculated the connectivity strength within both the negative and positive networks independently, generating two corresponding strength maps at the vertex level. In addition to these sub-networks, we calculated the connectivity strength for each vertex in a fully connected network that included all correlations, irrespective of their sign, without applying any thresholding, resulting in three distinct vertex-wise strength maps: negative, positive, and full.

### 2.8. Mapping Gradients and Connectivity Strength to Economo–Koskinas Cytoarchitecture

The Economo–Koskinas Cytoarchitecture classification system (Scholtens et al., 2018) is an important tool in the field of neuroscience for describing the structure of cerebral cortex cells. The system was proposed by Constantin von Economo and George N. Koskinas in 1925 to divide cortical regions by detailed cellular structural features. To examine the relationship between gradients, MCN connectivity strength, and cortical cytoarchitecture, we mapped both the gradient information and vertex-wise MCN connectivity strength values onto the Economo–Koskinas parcellation. This parcellation, as included in the ENIGMA Toolbox (Lariviere et al., 2021), classifies the cortex into five distinct cytoarchitectonic types: agranular, frontal, parietal, polar, and granular. Below, we elaborate on each type and its functional significance: (i) Agranular: Characterized by sparse layers II and IV, this region lacks a prominent granular layer and is predominantly implicated in motor control and the planning and execution of movements. (ii) Frontal: Distinguished by discernible layers II and IV, this area plays a pivotal role in cognitive control, emotional regulation, and decision-making processes critical to behavior. (iii) Parietal: Featuring dense layers II and IV populated with small, slender pyramidal cells, this region is integral to spatial perception, motor coordination, attention, and memory functions. (iv) Polar: Marked by a thinner cortical profile yet elevated cellular density, particularly in granular layers, this type is associated with higher-order cognitive abilities, including advanced decision-making and complex information processing. (v) Granular (koniocortex): Defined by a thin structure rich in small cells, especially in layer IV, and a sparse layer V, this region exhibits intricate laminar organization and pronounced functional specialization (Fotiadis et al., 2023). Its development and integrity are crucial for advanced cognition and specialized sensory processing. For each cytoarchitectonic type, we calculated the mean MCN connectivity strength across all vertices within that type. This approach allowed us to investigate relations between gradient characteristics, MCN connectivity strength, and the structural organization of the cortex as defined by cytoarchitectonic features.

### 2.9. Cognitive Meta-Analysis

To further elucidate the relationship between gradients, MCN connectivity strength, and cognitive and behavioral functions, we employed BrainStat toolbox (Lariviere et al., 2023) for a meta-analytic decoding analysis (Yarkoni et al., 2011). The BrainStat toolbox (https://github.com/MICA-MNI/BrainStat, accessed on 1 January 2024) utilized pre-computed Neurosynth maps encompassing 3228 terms derived from the latest Neurosynth database (version-7) using the vocab-terms annotation approach (https://github.com/neurosynth/neurosynth-data, accessed on 1 January 2024). To identify terms most closely associated with gradients and MCN connectivity strength, we conducted the following analysis. First, we employed a surface decoder to interpolate data from the cortical surface to voxels within the gray matter volume, specifically between the pial and white matter surfaces. Next, using only voxels with non-zero values in both this interpolated volume and the Neurosynth database, we computed Pearson correlations for each feature (i.e., term).

### 2.10. Correlation of Gradients and MCN Connectivity with Multimodal Cortical Features

To investigate the relationships between gradients and multimodal cortical features, as well as the relationships between MCN connectivity and multimodal cortical features (Luo et al., 2024; Sydnor et al., 2021), we conducted vertex-wise correlation analyses. Specifically, we performed two distinct sets of correlations: one between gradients and 10 multimodal cortical features, and another between MCN connectivity strength and the same 10 features. These multimodal cortical features included anatomical hierarchy (Glasser & Van Essen, 2011), functional hierarchy (Margulies et al., 2016), evolutionary hierarchy (Hill et al., 2010), allometric scaling (Reardon et al., 2018), aerobic glycolysis (Vaishnavi et al., 2010), cerebral blood flow (Satterthwaite et al., 2014), gene expression (Burt et al., 2018), NeuroSynth (quantified by the first principal component of NeuroSynth meta-analytic decodings) (Yarkoni et al., 2011), externopyramidization (Paquola et al., 2020), and cortical thickness (quantified from structural MRI, Human Connectome Project S1200 data). All data files listed in Table 1 can be downloaded from the official GitHub repository for the project (https://github.com/PennLINC/S-A_ArchetypalAxis/tree/main/FSLRVertex, accessed on 1 January 2024) (Sydnor et al., 2021). By calculating correlations across the entire cortical surface for each set, we aimed to uncover distinct associations between gradients and these features, as well as between MCN connectivity strength and the same features. Statistical significance for both sets of correlations was determined using Bonferroni correction to account for multiple comparisons.

### 2.11. Higher-Order Landscape in Vertex-Wise MCN

To explore the higher-order topological landscape of MCN, we went beyond the node and edge levels and employed graphlets analysis to count subgraphs with 3, 4, and 5 nodes (Hočevar & Demčar, 2016; Przulj, 2007). Specifically, we selected four specific correlation thresholds: −0.4, −0.2, 0.2, and 0.4. For negative thresholds (−0.4 and −0.2), we retained all edges with correlations less than or equal to these values, generating the corresponding binary networks. For positive thresholds (0.2 and 0.4), we retained all edges with correlations greater than or equal to these values, similarly generating the corresponding binary networks. This approach allowed us to systematically examine the distribution of 30 distinct topological graphlets (comprising 3-node, 4-node, and 5-node subgraphs) across different thresholds. We applied the MOSS algorithm (P. Wang et al., 2018), which is computationally efficient in approximating graphlet counts in large-scale networks, to compute the frequencies of the 30 graphlets in the thresholded binary networks. To facilitate comparison, we normalized the computed graphlet frequencies. Specifically, for each thresholded network, let $f\left(G_{i}\right)$ be the frequency of graphlets $G_{i}$ where $i\in \left(0\ldots 29\right)$. Then, we calculated the normalized frequency of each $f\left(G_{i}\right)$ by dividing it with $\sum_{i=0}^{29}f\left(G_{i}\right)$, to avoid the zero in the denominator, where we added 1 to each of the frequencies, and take the logarithm (10-base) of the normalized frequency$\;{f\left(G_{i}\right)}^{*}$, thus the normalized graphlets frequency matrix became,

$${f\left(G_{i}\right)}^{*}=\log\left(1+\frac{f\left(G_{i}\right)+1}{\sum_{i=0}^{29}\left(f\left(G_{i}\right)+1\right)}\right)$$

This resulted in the normalized graphlet frequency matrix. To maintain consistency in the graphlet analysis, we sorted the order of graphlets calculated by MOSS according to the graphlet ordering scheme defined by Przulj (Przulj, 2007). This alignment allowed for direct comparison of graphlet counts across methods.

**Figure 1:**
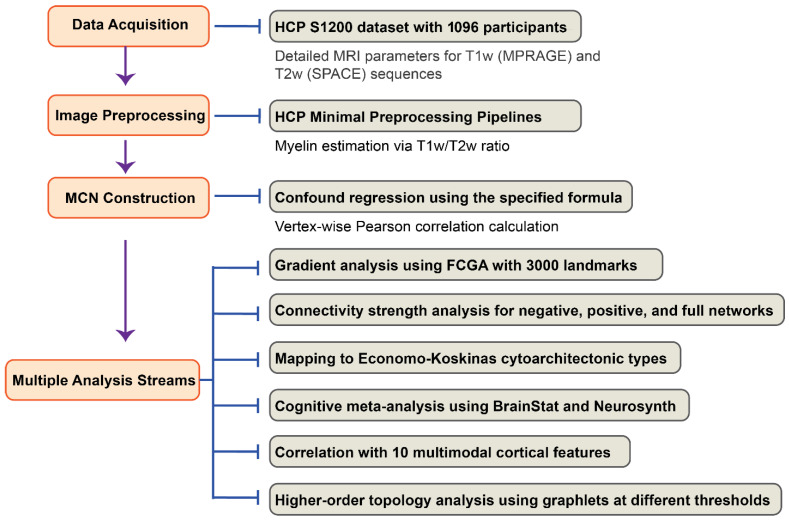
**Methodological Pipeline for Myelin Covariance Network Analysis**. This flowchart illustrates the comprehensive analytical pipeline for investigating cortical MCN using data from the Human Connectome Project. The analysis begins with acquisition of T1w MPRAGE and T2w SPACE neuroimaging data from 1096 healthy participants (age 22–37). Following HCP minimal preprocessing pipelines, myelin content was estimated using the T1w/T2w ratio method, which enhances cortical myelin contrast by approximating the squared myelin content. Vertex-wise MCNs were constructed after regressing out confounding variables (age, age^2^, gender, and age × gender interactions), yielding a 59,412 × 59,412 correlation matrix. Six parallel analytical approaches were then implemented: (1) gradient analysis using Fast Connectivity Gradient Approximation (FCGA) with 3000 random landmarks to extract principal components of connectivity; (2) connectivity strength analysis examining negative, positive, and full network connections; (3) mapping of gradients and connectivity strength to five Economo–Koskinas cytoarchitectonic types; (4) cognitive meta-analysis using the BrainStat toolbox and Neurosynth; (5) correlation analysis with 10 multimodal cortical features including anatomical hierarchy, functional hierarchy, and gene expression, etc.; and (6) higher-order topological analysis using graphlets at various correlation thresholds (−0.4, −0.2, 0.2, 0.4).

The flowchart of this study is shown in Figure 1.

## 3. Results

### 3.1. Gradients and MCN Connectivity Strength Maps

**Figure 2:**
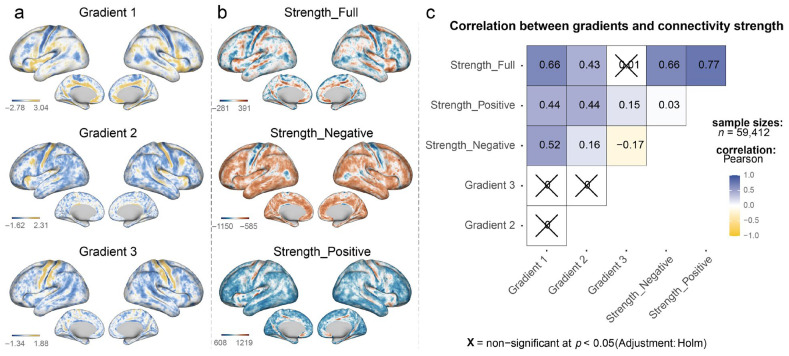
**Representations of gradient components, connectivity strength patterns, and their correlations in the MCN.** This figure presents the spatial patterns and relationships between principal gradients and connectivity strength measures in the vertex-wise MCN. (**a**) Brain surface renderings of the first three principal gradients (Gradient 1, Gradient 2, and Gradient 3) extracted using Fast Connectivity Gradient Approximation (FCGA). Color scales represent gradient values, with blue indicating lower values and yellow indicating higher values. (**b**) Brain surface renderings of three connectivity strength measures: full network strength (Strength_Full), negative correlation strength (Strength_Negative), and positive correlation strength (Strength_Positive). (**c**) Correlation matrix showing Pearson correlation coefficients between gradients and connectivity strength measures across all 59,412 vertices. The color scale ranges from dark blue (strong positive correlation) to white (no correlation) to yellow (negative correlation). Crossed-out cells (X) indicate non-significant correlations at *p* < 0.05 after Holm multiple comparison adjustment. Strong positive correlations exist between Gradient 1 and all strength measures, particularly with Strength_Full (r = 0.66), while Gradient 3 shows weak or non-significant correlations with strength measures.

The first three gradients collectively explained 28.27% of the variance in MCN. As subsequent gradients contributed less and to simplify statistical calculations, only the first three gradients were considered for downstream analyses. Gradient 1 to Gradient 3 explained 18.67%, 5.34%, and 4.25% of the variance, respectively. Subsequently, correlations between the three gradients and the connectivity strength of three vertex-level networks (negative, positive, and full) were calculated. The results showed that Gradient 1 was significantly positively correlated with Strength_Full (r = 0.66, *p*-value < 10^−6^), Strength_Positive (r = 0.44, *p*-value < 10^−6^), and Strength_Negative (r = 0.52, *p*-value < 10^−6^). For Gradient 2, correlations with Strength_Full and Strength_Positive were 0.43 and 0.44, respectively, all *p*-values were <10^−6^. Correlations of Gradient 3 with Strength_Positive and Strength_Negative were 0.15 and −0.17, respectively, all *p*-values were <10^−6^. All reported *p*-values were adjusted using the Holm-Bonferroni method for multiple comparisons (Figure 2).

### 3.2. Gradients and Connectivity Strength with Economo–Koskinas Cytoarchitectural Classes

**Figure 3:**
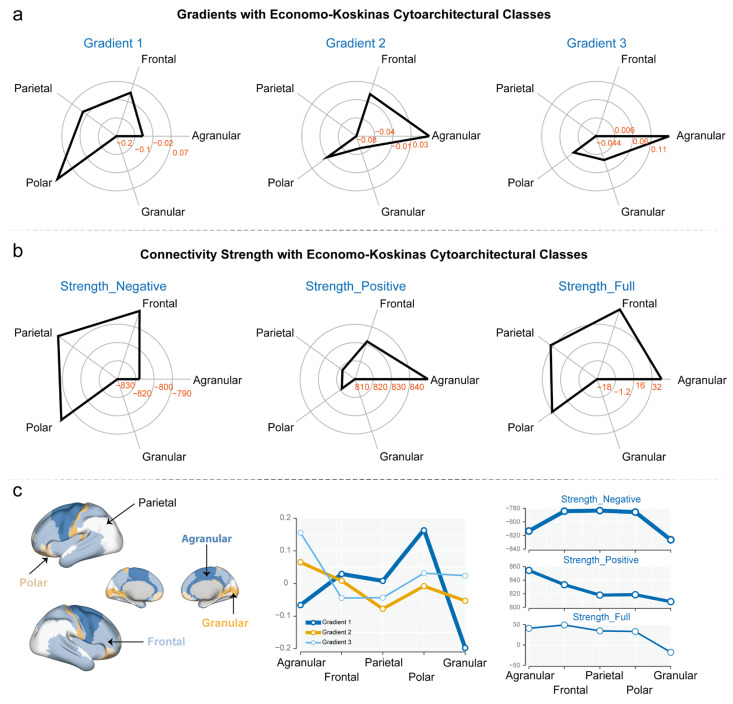
**Distribution of Myelin Covariance Network Properties Across Economo–Koskinas Cytoarchitectural Classes.** (**a**) Radar plots showing the distribution of three principal gradients of the MCN across five Economo–Koskinas cytoarchitectural classes (Agranular, Frontal, Parietal, Polar, and Granular). Gradient 1 shows highest values in Polar regions (0.1651) and lowest in Granular regions (−0.1975). Gradient 2 shows highest values in Agranular regions (0.0654) and lowest in Parietal regions (−0.0774). Gradient 3 shows highest values in Agranular regions (0.1558) and lowest in Frontal regions (−0.0443). (**b**) Radar plots illustrating connectivity strength distributions across the same cytoarchitectural classes for three different network types. Strength_Negative shows strongest negative connections in Granular regions (−826.32). Strength_Positive shows strongest positive connections in Agranular regions (854.33). Strength_Full (combined positive and negative connections) shows highest values in Frontal regions (49.07) and lowest (negative) values in Granular regions (−17.97). (**c**) Left panel: Brain surface renderings highlighting the anatomical locations of the five Economo–Koskinas cytoarchitectural classes. Middle panel: Line graph comparing the distribution patterns of the three principal gradients across cytoarchitectural classes. Right panel: Line graphs showing the distribution patterns of the three connectivity strength measures across cytoarchitectural classes.

We mapped the vertices of the MCN onto the Economo–Koskinas cytoarchitectural classes and analyzed their distribution within the Agranular, Frontal, Parietal, Polar, and Granular categories. The results showed that the distribution of *gradient 1* across the 5 categories was: Agranular (−0.0659), Frontal (0.0296), Parietal (0.0085), Polar (0.1651), and Granular (−0.1975). The standard deviation of gradient 1’s distribution across the 5 categories was 0.1331. The distribution of *gradient 2* across the 5 categories was as follows: Agranular (0.0654), Frontal (0.0091), Parietal (−0.0774), Polar (−0.0077), and Granular (−0.0528). The standard deviation of gradient 2’s distribution across the 5 categories was 0.0557. The distribution of *gradient 3* across the 5 categories was: Agranular (0.1558), Frontal (−0.0443), Parietal (−0.0433), Polar (0.0322), and Granular (0.0244). The standard deviation of gradient 3’s distribution across the 5 categories was 0.0816. Overall, gradient 1 exhibited the largest variability across the 5 categories, followed by gradient 3, with gradient 2 showing the smallest variability. In Gradient 1, the Polar category (0.1651) was dominant, while in Gradients 2 and 3, the Agranular category occupied the primary position (Figure 3a).

The results showed the connectivity strength of three vertex-level networks (negative, positive, and full) distributed across the five Economo–Koskinas cytoarchitectural classes: Agranular, Frontal, Parietal, Polar, and Granular. The distribution of *Strength_Negative* across the 5 categories is: Agranular (−813.40), Frontal (−784.22), Parietal (−783.29), Polar (−785.57), and Granular (−826.32), with a standard deviation of 19.99. The distribution of *Strength_Positive* across the 5 categories is: Agranular (854.33), Frontal (833.29), Parietal (817.99), Polar (818.56), and Granular (808.35), with a standard deviation of 17.93. The distribution of *Strength_Full* across the 5 categories is: Agranular (40.93), Frontal (49.07), Parietal (34.70), Polar (32.99), and Granular (−17.97), with a standard deviation of 26.43 (Figure 3b).

### 3.3. Interpreting Gradients and Connectivity Strength in the Context of Cognitive Maps

**Figure 4:**
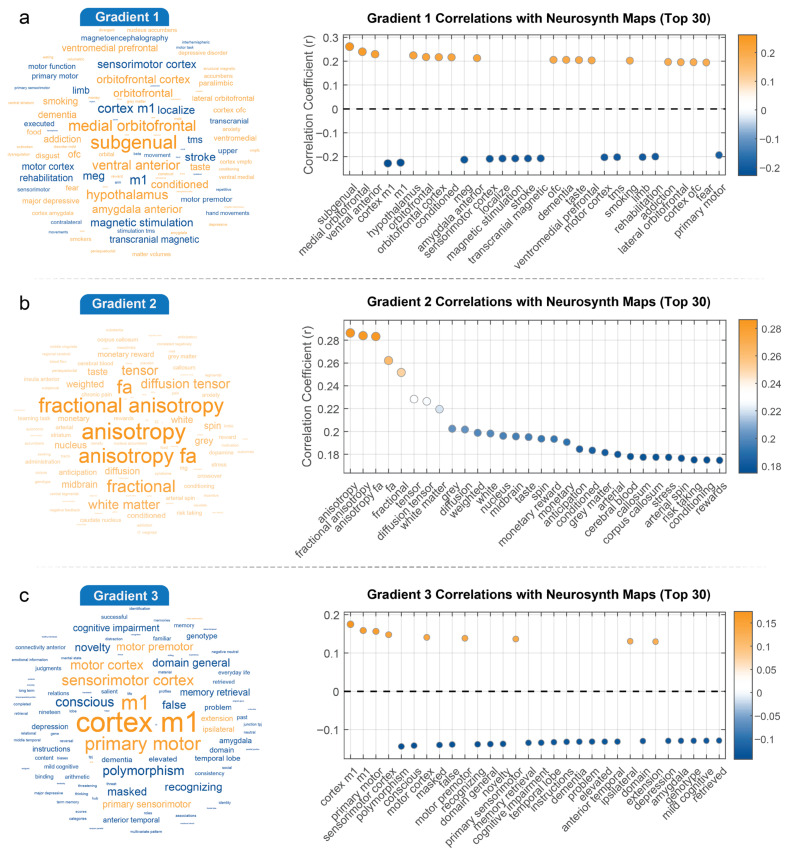
**Correlations Between Principal Myelin Covariance Gradients and Neurosynth Meta-Analysis Maps.** The figure displays correlations between three principal myelin covariance gradients and Neurosynth meta-analysis maps, with both word cloud visualizations (left) and correlation plots (right) showing the top 30 correlations for each gradient. (**a**) Gradient 1 (top panel) shows a functional dissociation between systems. Positive correlations (orange) appear with limbic and affective processing regions, with strongest associations to medial prefrontal, ventromedial prefrontal, and orbitofrontal cortices (r ≈ 0.22–0.25). Terms related to subgenual cortex, ventral anterior regions, hypothalamus, and amygdala also show robust positive correlations. In contrast, negative correlations (blue, r ≈ −0.2) are observed with sensorimotor systems, including primary motor, sensorimotor cortex, motor function, and rehabilitation terms. (**b**) Gradient 2 (middle panel) primarily relates to white matter microstructural properties, with strongest positive correlations (orange-yellow, r ≈ 0.28–0.29) with fractional anisotropy measures, diffusion tensor imaging metrics, and white matter properties. The correlation strength gradually decreases across the 30 top terms (r = 0.29 to 0.17), with no strong negative correlations observed, suggesting this gradient primarily captures variations in white matter organization patterns. (**c**) Gradient 3 (bottom panel) reveals another functional dissociation. Positive correlations (orange-yellow, r ≈ 0.13–0.18) are observed with motor system terms including cortex m1, primary motor, sensorimotor cortex, and motor premotor areas. Negative correlations (blue, r ≈ −0.12 to −0.14) are seen with terms related to higher cognitive functions and pathological states, including cognitive impairment, memory retrieval, novelty, dementia, and polymorphism, suggesting this gradient may distinguish between basic sensorimotor functions and higher-order cognitive processes.

**Figure 5:**
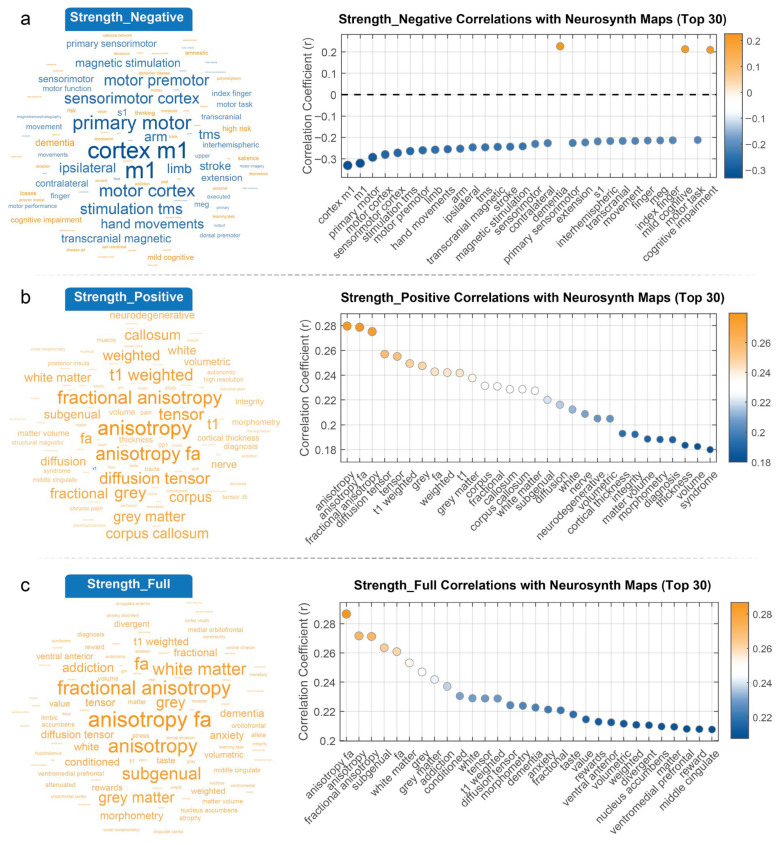
**Correlations Between Myelin Covariance Network Connectivity Strength and Neurosynth Meta-Analysis Maps.** This figure illustrates the correlations between three different MCN connectivity strength measures (Negative, Positive, and Full) and Neurosynth meta-analysis terms, presented as word clouds (left) and correlation plots (right) showing the top 30 correlations for each strength measure. (**a**) Strength_Negative (top panel) demonstrates predominantly negative correlations (blue, r ≈ −0.21 to −0.32) with motor and sensorimotor systems. The strongest negative correlations appear with cortex m1, primary motor, sensorimotor cortex, motor premotor, and motor cortex terms. Interestingly, a few terms show positive correlations (orange, r ≈ 0.2), including dementia and cognitive impairment, suggesting this network may capture an inverse relationship between motor function and neurodegenerative conditions. (**b**) Strength_Positive (middle panel) shows robust positive correlations (orange-yellow, r ≈ 0.28–0.30) with white matter microstructural properties. The most strongly correlated terms include fractional anisotropy, anisotropy fa, diffusion tensor, t1 weighted, white matter, corpus callosum, and gray matter. This pattern suggests that positive connectivity strength in the MCN relates primarily to white matter integrity and structural connectivity metrics derived from diffusion tensor imaging. (**c**) Strength_Full (bottom panel), representing the combined positive and negative connectivity, reveals a pattern similar to Strength_Positive but with some additional terms. The strongest correlations (orange-yellow, r ≈ 0.27–0.29) are with fractional anisotropy, anisotropy fa, and white matter structural terms. Additionally, terms related to affective and cognitive processes such as subgenual, addiction, anxiety, and dementia show moderate positive correlations, indicating that the full connectivity strength measure captures both structural and functional dimensions of brain organization.

We assessed the correlations of both the gradients and the three vertex-level networks (negative, positive, and full) with cognitive meta-analysis terms separately, visualizing the results in word clouds (Figure 4 and Figure 5). In these word clouds, the font size of each term reflects the absolute value of the correlation, with larger fonts indicating stronger correlations, while the color denotes the direction of the correlation (orange for positive correlations, blue for negative correlations). The results for each gradient are described below.

Gradient 1 strongly differentiates between brain regions involved in affective processing and regulation (positive correlation, orange-yellow, larger font), including subgenual prefrontal cortex, medial orbitofrontal cortex, ventral anterior regions, hypothalamus, orbitofrontal cortex (OFC), and amygdala anterior. In contrast, regions dedicated to motor control and sensorimotor functions (negative correlation, blue, larger font), such as motor cortex, sensorimotor cortex, and specifically the primary motor cortex (cortex m1), show a strong negative correlation (Figure 4a). Gradient 2 is primarily characterized by strong positive correlations (orange-yellow, larger font) among terms related to white matter microstructure, most notably fractional anisotropy (FA), including fractional anisotropy, anisotropy, anisotropy fa, diffusion tensor, and white matter (Figure 4b). There are no prominently large blue terms indicating strong negative correlations. This suggests that gradient 2 mainly captures a pattern of myelin covariance related to the integrity and organization of white matter tracts across the brain. Gradient 3 clearly contrasts brain regions showing positive correlations (orange-yellow), including cortex m1, m1, primary motor, sensorimotor cortex, and motor cortex (Figure 4c). These terms unequivocally point to areas directly involved in motor control and sensory-motor processing. Conversely, the gradient shows negative correlations (blue) with terms such as cognitive impairment, memory retrieval, novelty, domain general, recognizing, dementia, mild cognitive, genotype, and polymorphism. This suggests that gradient 3 might reflect a myelin covariance pattern that distinguishes between motor regions and areas involved in higher-level cognitive functions, potentially highlighting differences related to cognitive abilities, neurological states, or even underlying genetic variations.

*Strength_Negative* (Figure 5a), terms related to motor and sensory functions were prominent. Most of the correlations were negative, shown in blue. These terms included the primary motor cortex, M1, motor cortex, and sensorimotor, hand movements, finger, task, and transcranial magnetic stimulation (TMS). It is remarkable that terms like dementia, depression, and Alzheimer’s disease showed up with moderate positive correlations, indicating the Strength_Negative network may be associated with neurodegenerative or psychiatric conditions. For *Strength_Positive* (Figure 5b, Middle), the word cloud was mainly composed of terms related to white matter microstructure, and these had positive correlations. The terms included fractional anisotropy, white matter, T1 weighted, and diffusion tensor. This implies that Strength_Positive is strongly linked to white matter integrity and structural connectivity. Other positively correlated terms were neurodegenerative, morphometry, cortical thickness, and matter volume, highlighting the role that *Strength_Positive* networks play in structural characteristics. *Strength_Full* (Figure 5c) displayed the most prominent terms included anisotropy fa, fractional anisotropy, subgenual, white matter, and gray matter, all showing strong positive correlations (orange), consistent with the positive network’s emphasis on white matter microstructure. However, cognitive and psychiatric terms such as addiction, dementia, anxiety disorders, and rewards also appeared with strong positive correlations, indicating that the *Strength_Full* captures both structural and cognitive dimensions.

### 3.4. Gradients and MCN Connectivity with Multimodal Cortical Features

We investigated the relationships between MCNs and cortical features by computing Pearson correlation coefficients between three gradients (Gradient 1, Gradient 2, and Gradient 3) and three strength networks (Strength-Negative, Strength-Positive, and Strength-Full) with 10 cortical maps: Anatomical Hierarchy (AH), Functional Hierarchy (FH), Evolutionary Hierarchy (EH), Allometric Scaling (AS), Aerobic Glycolysis (AG), Cerebral Blood Flow (CB), Gene Expression (GE), NeuroSynth (NS), Externopyramidization (EX), and Cortical Thickness (CT). The results are visualized in two correlation coefficient heatmaps, with color intensity indicating the strength and direction of the correlation (orange for positive, blue for negative), and an “X” denoting non-significant correlations after Bonferroni correction (*p* > 0.05/30).

**Figure 6:**
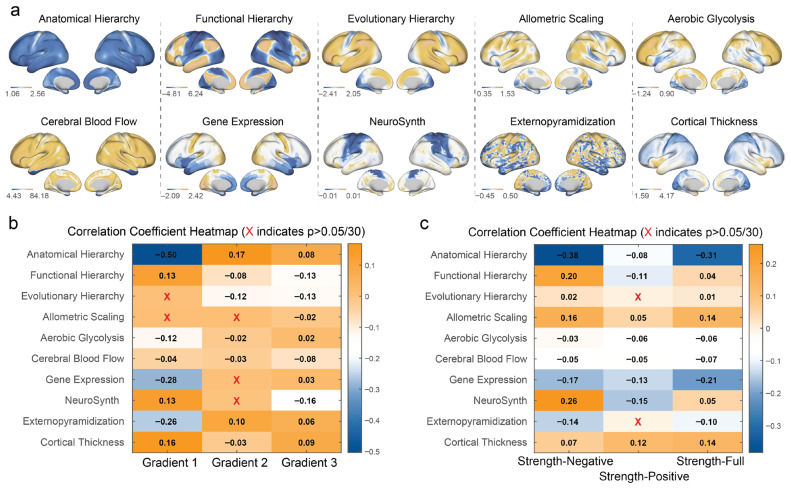
**Cortical feature correlations with myelin covariance network gradients and strength measures.** (**a**) Brain surface renderings of ten cortical features: Anatomical Hierarchy, Functional Hierarchy, Evolutionary Hierarchy, Allometric Scaling, Aerobic Glycolysis (top row); Cerebral Blood Flow, Gene Expression, NeuroSynth, Externopyramidization, and Cortical Thickness (bottom row). Each pair shows lateral and medial views with corresponding value ranges below. (**b**) Correlation coefficient heatmap between three MCN gradients (Gradient 1, Gradient 2, Gradient 3) and the ten cortical features. Gradient 1 exhibited the strongest correlations, notably with Anatomical Hierarchy (r = −0.50), Gene Expression (r = −0.28), and Externopyramidization (r = −0.26). (**c**) Correlation coefficient heatmap between three MCN strength networks (Strength-Negative, Strength-Positive, Strength-Full) and the same cortical features. In both heatmaps, orange indicates positive correlations, blue indicates negative correlations, and “X” marks non-significant correlations after Bonferroni correction (*p* > 0.05/30). These findings suggest that MCNs capture distinct aspects of cortical organization.

Correlations with gradients. Gradient 1 showed the most pronounced correlations, ranging from −0.50 to 0.16. There was a significant negative association with Anatomical Hierarchy (−0.50). Significant negative correlations were also found with Gene Expression (−0.28) and Externopyramidization (−0.26), and positive correlations with Functional Hierarchy (0.13), NeuroSynth (0.13), and Cortical Thickness (0.16). Correlations with Evolutionary Hierarchy and Allometric Scaling were not significant. Gradient 2 exhibited weaker correlations overall (−0.12 to 0.17), with the strongest being a positive correlation with Anatomical Hierarchy (0.17). Several correlations were not significant. Gradient 3 showed correlations ranging from −0.16 to 0.09, with the strongest being a negative correlation with NeuroSynth (−0.16). Other notable correlations included negative associations with Functional Hierarchy (−0.13) and Evolutionary Hierarchy (−0.13), and a positive correlation with Cortical Thickness (0.09); see Figure 6a.

Correlations with connectivity strength. The Strength-Negative network exhibited the strongest overall correlations, ranging from −0.38 to 0.26. Notably, it showed a significant negative correlation with Anatomical Hierarchy (−0.38), suggesting lower strength-negative is associated with higher anatomical hierarchy. Significant negative correlations were also found with Gene Expression (−0.17) and Externopyramidization (−0.14), while positive correlations were observed with Functional Hierarchy (0.20), Allometric Scaling (0.16), and NeuroSynth (0.26). The Strength-Positive network showed weaker correlations, ranging from −0.15 to 0.12, with the strongest being a negative association with NeuroSynth (−0.15). Several correlations were not significant. The Strength-Full network displayed correlations ranging from −0.31 to 0.14, with significant negative correlations with Anatomical Hierarchy (−0.31) and Gene Expression (−0.21), and positive correlations with Allometric Scaling (0.14) and Cortical Thickness (0.14); see Figure 6b.

Comparative Insights across Gradient and Strength Analyses. Both Gradient 1 and Strength-Negative exhibited strong negative correlations with Anatomical Hierarchy (AH; −0.50 and −0.38, respectively), suggesting that both metrics, derived from structural MCNs, capture patterns of myelin organization that are inversely related to anatomical hierarchy, as measured by the T1/T2 ratio. However, Gradient 1 showed a stronger correlation (absolute value) with AH than Strength-Negative, indicating that the primary gradient of myelin covariance may more robustly reflect this relationship. In contrast, Gradient 2 and Strength-Positive both showed weaker correlations overall, with some failing to reach statistical significance, suggesting that these metrics may capture more subtle or less consistent patterns of myelin covariance in relation to the cortical maps. Gradient 3 and Strength-Full displayed more balanced correlation profiles, with Strength-Full showing stronger correlations with Allometric Scaling (AS; 0.14) and Cortical Thickness (CT; 0.14) compared to Gradient 3 (−0.02 and 0.09, respectively). Notably, Strength-Negative also showed a stronger positive correlation with NeuroSynth (NS; 0.26) compared to Gradient 1 (0.13), suggesting a more pronounced association with cognitive processes, as captured by NeuroSynth meta-analytic decodings, in the strength-based myelin covariance analysis. Evolutionary Hierarchy (EH) consistently showed the weakest correlations across both analyses, with non-significant results in some cases, suggesting that evolutionary cortical expansion may not be strongly related to the structural patterns of myelin covariance captured by either gradients or strength metrics in this context.

### 3.5. Higher-Order Topological Landscape of MCN

To explore the higher-order topological organization of the MCN, we conducted a graphlet analysis, examining the prevalence of 3-node, 4-node, and 5-node subgraphs (30 distinct graphlets) at four different correlation thresholds: −0.4, −0.2, 0.2, and 0.4. For each threshold, binary networks were constructed, and the normalized frequencies of the 30 graphlets were computed.

Graphlet distribution across thresholds: The graphlet analysis revealed distinct patterns of subgraph prevalence across the different correlation thresholds. Negative Threshold of −0.4: at this stringent negative threshold, the most frequent graphlets were G11 (normalized frequency = 0.1367), followed by G10 (0.0839), G16 (0.0701), and G20 (0.0514). Negative Threshold of −0.2: when the negative threshold was relaxed to −0.2, the most prevalent graphlet remained G11, with an increased normalized frequency of 0.1873. Other relatively frequent graphlets at this threshold included G10 (0.0649), G16 (0.0570), and G20 (0.0378). Positive Threshold of 0.2: at the positive threshold of 0.2, the graphlet distribution shifted. The most frequent graphlets were G09 (0.0782), G13 (0.0666), G10 (0.0641), and G12 (0.0391). Positive Threshold of 0.4: at the higher positive threshold of 0.4, the most frequent graphlets were G09 (0.0850), G13 (0.0712), G10 (0.0387), and G12 (0.0386).

**Figure 7:**
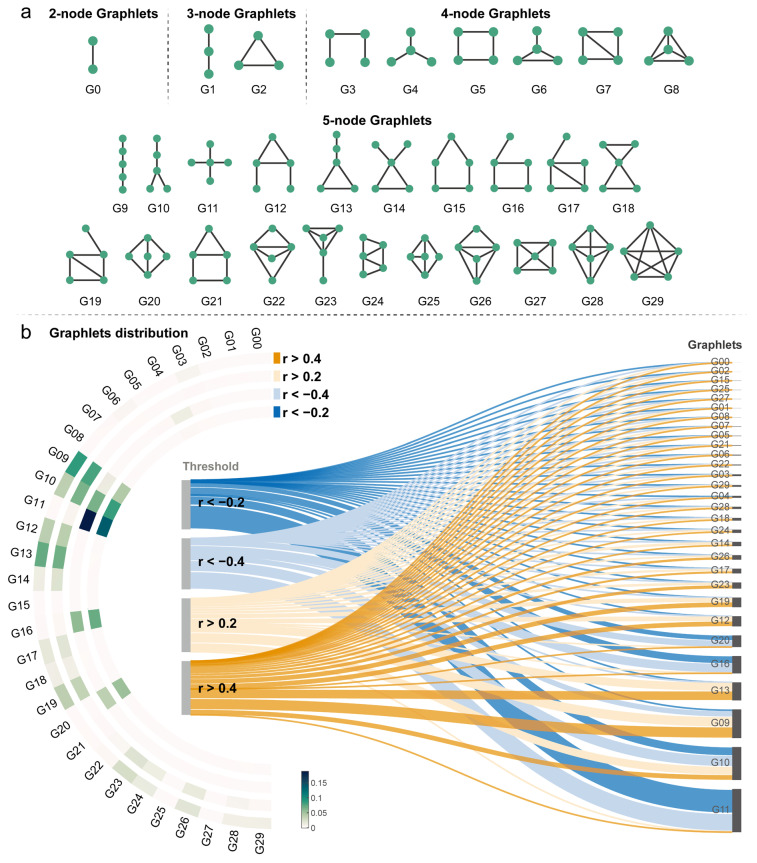
**Higher-order topological organization of myelin covariance networks through graphlet analysis**. (**a**) Visualization of 30 distinct graphlets (G0–G29) representing all possible connected subgraphs with 2–5 nodes. Each graphlet captures a specific local connectivity pattern. (**b**) Distribution of graphlets across four correlation thresholds (r > 0.4, r > 0.2, r < −0.2, r < −0.4) shown as a flow diagram. Line thickness represents the normalized frequency of each graphlet, with color indicating the corresponding threshold. The circular heatmap on the left displays normalized graphlet frequencies (darker green indicating higher frequency). Notable patterns emerge where star-like structures (particularly G10, G11) dominate negative correlations, while path-like and triangular structures (G09, G13) predominate in positive correlations. This analysis reveals fundamentally different topological organizations between positively and negatively correlated components of the MCN, suggesting distinct underlying biological mechanisms governing these relationships.

Comparison of graphlet frequencies across thresholds: Several interesting trends emerge when comparing graphlet frequencies across different thresholds. Graphlet G11 and G10 (star-like graphlet) showed the higher frequencies at both negative thresholds, suggesting their importance in the topology of negatively correlated edges. In contrast, graphlets G09 (path-like graphlet) and G13 (triangle and path) exhibited the highest frequencies at both positive thresholds, indicating their prominence in positively correlated subnetworks. Notably, the frequency of G11 decreased substantially at positive thresholds, while the frequencies of G09 and G13 were low at negative thresholds, highlighting the distinct topological characteristics of positively and negatively correlated parts of the MCN (Figure 7).

## 4. Discussion

The present study aimed to provide a comprehensive, vertex-level characterization of human cortical myelin covariance networks (MCNs) by identifying their principal gradients, cognitive associations, and higher-order topological organization. Our results successfully addressed these objectives, providing new insights into the structural organization of cortical myelin. Our core finding, in relation to our first objective, is that the principal gradient (Gradient 1) is spatially aligned with the axis extending from the sensory-motor cortex to the association cortex, consistent with existing cortical hierarchy models. Addressing our second objective, this gradient was strongly negatively correlated with anatomical hierarchy (r = −0.50) and closely coupled with network connectivity strength (r = 0.66 with Strength_Full). Furthermore, Gradient 1 exhibited a clear functional dissociation between affective processing (positive correlation) and sensorimotor systems (negative correlation). Our findings also linked gray matter myelin patterns to white matter microstructure, as Gradient 2 showed robust associations with fractional anisotropy (FA). Finally, addressing our third objective, the graphlet analysis revealed a fundamental topological distinction: negative covariance connections were dominated by star-like structures (G10, G11), while positive connections were dominated by path-like and triangular structures (G09, G13). These findings confirm our hypothesis that the observed topological differences are not accidental but are instead embedded within the common organizational principles of the human cortex.

Our extensive analysis of MCNs offers new insights into the structural organization of human cortical myelin. The application of a gradient decomposition analysis yielded three major gradients that accounted for a total of 28.27% of the variance in the MCN—of which Gradient 1 explained 18.67% of the variance. Each of these gradients showed very different relationships with cytoarchitectural classes, cognitive functions, and multimodal cortical properties. This difference suggests that MCNs contain fundamental aspects of brain organization in addition to already established neuroimaging metrics.

The primary gradient (Gradient 1) had a moderately strong negative correlation with anatomical hierarchy (−0.50), gene expression (−0.28), and externopyramidization (−0.26). This is consistent with the previous literature that has identified an inverse relationship between cortical myelin and cortical hierarchy (Huntenburg et al., 2018; Wagstyl et al., 2015). The strong relationship between Gradient 1 and connectivity strength (particularly Strength_Full, r = 0.66) indicates that Gradient 1 can succinctly describe the overall connectivity characteristics of the MCN. The functional dissociation that Gradient 1 presented (affective processing, positive correlations vs. sensorimotor systems, negative correlations), is also in line with the existing hierarchical models of cortical organization (Badre & D’Esposito, 2007; Margulies et al., 2016). This dissociation mirrors the integration–segregation principle proposed by Mesulam (Mesulam, 1998), where unimodal sensory regions exhibit distinct connectivity patterns compared to transmodal association areas.

Gradient 2, explaining 5.34% of variance, demonstrated a strong association with white matter microstructural properties based on the Neurosynth meta-analysis. The robust positive correlations with fractional anisotropy and diffusion tensor imaging metrics suggest that this gradient may reflect the underlying white matter architecture supporting cortical myelin patterns (Grydeland et al., 2013; Rahmanzadeh et al., 2021). This finding provides an important link between gray matter myelin and white matter organization, potentially offering a more integrated view of brain structural connectivity than either measure alone.

Gradient 3 (4.25% of variance) revealed another functional dissociation between motor system regions and areas involved in higher cognitive functions. This separation is particularly intriguing as it may reflect evolutionary trade-offs between basic sensorimotor processing and higher-order cognition (Hill et al., 2010). The weak correlation of Gradient 3 with connectivity strength measures suggests it captures aspects of myelin covariance that are not simply related to connection density but rather to specific functional specializations.

The analysis of MCN properties across Economo–Koskinas cytoarchitectural classes provided further evidence for the biological relevance of these gradients. Gradient 1 showed the largest variability across cytoarchitectural classes, with highest values in Polar regions and lowest in Granular regions. This pattern corresponds well with known differences in myelin between these architecturally distinct regions. The preferential distribution of strength measures across cytoarchitectural classes—with Strength_Negative showing the strongest connections in Granular regions and Strength_Positive in Agranular regions—reveals a systematic relationship between myelin covariance and cortical cytoarchitecture, supporting earlier findings on structure–function relationships in the cortex (Burt et al., 2018).

Our graphlets analysis revealed qualitatively different topological organizations of the MCN’s positively and negatively correlated components. Graphlets of star-like graphs (G10, G11) in negative thresholds were far more dominant than either path-like or triangular graphlets (G09, G13). This notable difference suggests that positively and negatively correlated MCNs are governed by radically different underlying mechanisms. This topological differentiation might reflect different developmental processes shaping myelin patterns across the cortex (Khundrakpam et al., 2016). Recent work on developmental covariance networks has shown similar topological distinctions, suggesting that such higher-order structures may represent conserved organizational principles across different neurobiological dimensions (Sebenius et al., 2023; Vasa et al., 2018).

The associations between MCN measures (gradients and strength) and multimodal cortical characteristics provide relevant context when interpreting the biological importance of myelin covariance. Negative correlation between Gradient 1 and anatomical hierarchy and moderate correlations with gene expression and externopyramidization. It would seem that myelin covariance patterns are subject to some genetic and developmental processes that shape the differentiation of the cortex (Patel et al., 2019; Romero-Garcia et al., 2018), while the weaker correlations with evolutionary hierarchy for all MCN measures raise the interesting possibility that a greater proportion of variance in myelin covariance is driven by functional demands and inherent microstructural properties rather than evolutionary expansions (Buckner & Krienen, 2013).

The complementary patterns seen in gradient-based and strength-based analyses highlight the importance of using diverse analytical methods to study MCNs. Gradients reflect the main dimensions of variation in myelin covariance, while strength measures shed light on local connectivity patterns. Both methods show comparable connections to anatomical hierarchy and gene expression, but each uncovers distinct elements of how MCNs relate to cortical features.

Several limitations should be kept in mind when interpreting our results. First, our analyses focused on examining global covariance patterns using age as a statistical covariate, without constructing age-specific covariance networks; therefore, current research cannot make causal inferences. Second, our group-level approach may obscure meaningful individual variability and a key future direction is the development of robust methods for constructing and interpreting single-participant myelin covariance networks, which would be essential for clinical applications and personalized medicine. Third. we used the T1w/T2w ratio as an indirect measure of myelin content, a common approach but one with recognized limitations in terms of sensitivity and specificity (van der Weijden et al., 2021). Future studies could use alternative imaging techniques, like quantitative T1 mapping or magnetization transfer ratio (Weiskopf et al., 2021), gradient echo myelin water imaging (Shin et al., 2019), quantitative susceptibility mapping (QSM) and multi-echo T2 myelin water fraction (MWF) mapping techniques (Sandrone et al., 2023) to obtain more precise measurements of myelin. Fourth, our focus on group-level MCNs has offered useful insights, but it also comes with a trade-off—we may be missing important individual differences in myelin organization that could matter, especially in clinical contexts. Figuring out how to build and interpret MCNs at the individual level seems like an important next step, particularly if we want to move toward more personalized approaches in neuroimaging (Li et al., 2021; Wu et al., 2018, 2024). It might also be valuable to look at how MCN patterns relate to functional connectivity. Combining these two perspectives could help us understand not just how the brain is wired, but how that wiring supports (or limits) brain dynamics in real time. Finally, while our graphlet analysis pointed to clear differences in the topology of positive and negative MCN components, the biological reasons behind these patterns are still unclear. There is a lot of potential in bringing in other types of data (like genetics, molecular markers, or cellular-level information) to better understand what is driving these differences (Fornito et al., 2019).

## 5. Conclusions

This study constructed and characterized the systematic differences in gradients and network topology of cortical myelin covariance networks (MCNs) at the vertex level in a healthy adult sample, providing new insights into the structural organization of the human cortical myelin. Our core finding is that the principal gradient (Gradient 1) is spatially aligned with the axis extending from the sensory-motor cortex to the association cortex, which is consistent with existing models of cortical hierarchical organization. This gradient showed a significant negative correlation with anatomical hierarchy and was closely coupled with network connectivity strength. Furthermore, both the gradients and the connectivity strength of the covariance networks were found to be significantly correlated with the brain’s cognitive functions; for example, Gradient 1 exhibited a functional dissociation between affective processing and sensorimotor systems. More importantly, the positive and negative covariance networks exhibited significantly different topological structures: negative correlations were dominated by star-like structures, while positive correlations were dominated by path-like and triangular structures. This suggests that the topological differences we observed are not accidental but are instead embedded within the common organizational principles of the human cortex, from low-level (sensory-motor) to high-level (association) processing.

## Figures and Tables

**Table 1:** Summary of Multimodal Cortical Features. The table lists the ten neurobiological maps used in the correlation analyses, along with their descriptions and corresponding data filenames. All data are publicly available and were sourced from the study by (Sydnor et al., 2021), as detailed in the Data Availability statement.

Feature Name	Description	Corresponding GitHub Filename
**Anatomical Hierarchy**	A measure of hierarchical organization (often proxied by T1w/T2w myelination maps).	T1T2ratio.dscalar.nii
**Functional Hierarchy**	The principal gradient (G1) of functional connectivity variation across the cortex.	G1.fMRI.dscalar.nii
**Evolutionary Hierarchy**	A map reflecting the evolutionary expansion of the cerebral cortex.	Evolution.Expansion.dscalar.nii
**Allometric Scaling**	A measure related to how brain properties scale with brain size.	AllometricScaling.PNC20mm.dscalar.nii
**Aerobic Glycolysis**	A metabolic map indicating the rate of aerobic glycolysis, measured via PET.	PET.AG.dscalar.nii
**Cerebral Blood Flow**	A map of resting-state cerebral blood flow (CBF).	CBF.dscalar.nii
**Gene Expression**	The first principal component (PC1) of cortical gene expression from the Allen Human Brain Atlas (AHBA).	PC1.AHBA.dscalar.nii
**NeuroSynth**	The first principal component (PC1) of NeuroSynth meta-analytic decodings.	PC1.Neurosynth.dscalar.nii
**Externopyramidization**	A microstructural measure related to cytoarchitecture, often studied using high-resolution histology like the BigBrain dataset.	BigBrain.Histology.dscalar.nii
**Cortical Thickness**	A structural measure of the thickness of the cerebral cortex.	Cortical.Thickness.dscalar.nii

## Data Availability

The original data presented in the study are openly available. The MRI data from the Human Connectome Project (HCP, https://www.humanconnectome.org), a consortium led by Washington University, the University of Minnesota, and Oxford University (WU-Minn HCP). We downloaded the 1200 Subjects Group Average Data, released 1 August 2017 (https://www.humanconnectome.org/study/hcp-young-adult/article/s1200-group-average-data-release, accessed on 1 January 2024). The Multimodal Cortical Features data are available on GitHub at https://github.com/PennLINC/S-A_ArchetypalAxis/tree/main/FSLRVertex, accessed on 1 January 2024.
